# On the absolute photoionization cross section and threshold photoelectron spectrum of two reactive ketenes in lignin valorization: fulvenone and 2-carbonyl cyclohexadienone†

**DOI:** 10.1039/d1cp05206c

**Published:** 2022-01-26

**Authors:** Zeyou Pan, Andras Bodi, Jeroen A. van Bokhoven, Patrick Hemberger

**Affiliations:** Zeyou Pan, Andras Bodi, Jeroen A. van Bokhoven and Patrick Hemberger, Paul Scherrer Institute 5232 Villigen Switzerland patrick.hemberger@psi.ch; Zeyou Pan and Jeroen A. van Bokhoven, Institute for Chemical and Bioengineering, Department of Chemistry and Applied Biosciences, ETH Zurich 8093 Zurich Switzerland

## Abstract

We report the absolute photoionization cross section (PICS) of fulvenone and 2-carbonyl cyclohexadienone, two crucial ketene intermediates in lignin pyrolysis, combustion and organic synthesis. Both species were generated *in situ* by pyrolyzing salicylamide and dectected *via* imaging photoelectron photoion coincidence spectroscopy. In a deamination reaction, salicylamide loses ammonia yielding 2-carbonyl cyclohexadienone, a ketoketene, which further decarbonylates at higher pyrolysis temperatures to form fulvenone. We recorded the threshold photoelectron spectrum of the ketoketene and assigned the ground state (X̃^+2^A′′ ← X̃^1^A′) and excited state (Ã^+2^A′ ← X̃^1^A′) bands with the help of Franck–Condon simulations. Adiabatic ionization energies are 8.35 ± 0.01 and 9.19 ± 0.01 eV. In a minor reaction channel, the ketoketene isomerizes to benzpropiolactone, which decomposes subsequently to benzyne by CO_2_ loss. Potential energy surface and RRKM rate constant calculations agree with our experimental observations that the decarbonylation to fulvenone outcompetes the decarboxylation to benzyne by almost two orders of magnitude. The absolute PICS of fulvenone at 10.48 eV was determined to be 18.8 ± 3.8 Mb using NH_3_ as a calibrant. The PICS of 2-carbonyl cyclohexadienone was found to be 21.5 ± 8.6 Mb at 9 eV. Our PICS measument will enable the quantification of reactive ketenes in lignin valorization and combustion processes using photoionization techniques and provide advanced mechanistic and kinetics insights to aid the bottom-up optimization of such processes.

## Abstract 2

The absolute photoionization cross section (PICS) of these crucial ketene intermediates supports their quantification in lignin pyrolysis, combustion and organic synthesis.

## Graphical abstract

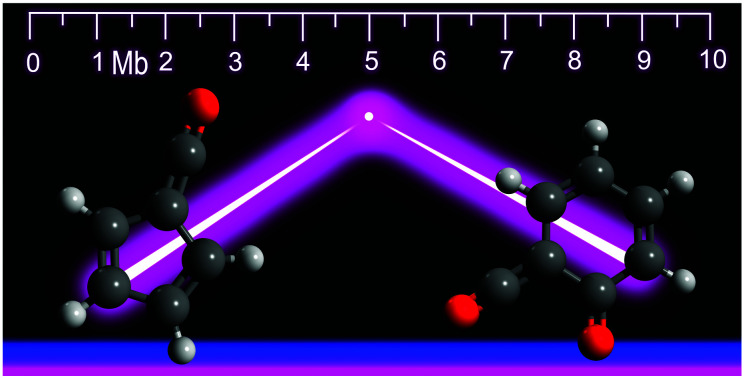

## Introduction

Fulvenone (c-C_5_H_4_=C=O), as a reactive ketene species, is not only a synthon in organic synthesis,^1–3^ for example in the cycloaddition of alkynes to yield functionalized arenes in medicinal chemistry, but was also observed as a reactive intermediate playing a critical role in combustion,^4,5^ pyrolysis^6,7^ and photochemistry.^8,9^ Reva *et al.* discovered fulvenone as a common product in UV-induced photochemistry of 2-isocyanophenol and 2-cyanophenol, nuclei of numerous compounds with important biological, pharmacological, and photophysical properties.^9^ In combustion chemistry, Wiersum pointed out that fulvenone can decarbonylate yielding the soot precursor C_5_H_4_.^4^ Recently, Bierkandt *et al.* detected the ketene in an anisole flame.^5^ We observed fulvenone as the central intermediate in guaiacol and catechol catalytic pyrolysis by imaging photoelectron photoion coincidence (iPEPICO) detection, which combines photoionization mass spectrometry (PIMS) and photoion mass-selected threshold photoelectron spectroscopy (ms-TPES).^7,10^

Due to its high reactivity, fulvenone evades detection using standard chemical analysis tools, such as GC/MS and NMR, which is the reason why fulvenone was only observed using photoionization mass spectrometry (PIMS), photoelectron spectroscopy (PES), matrix infrared spectroscopy (IR) and PEPICO detection.^7,11–13^ Most recently, Genossar *et al.* recorded the IR spectrum of fulvenone produced by salicylaldehyde pyrolysis.^13^ This ketene was also synthesized *in situ via* pyrolysis of lignin model compounds and characterized by photoelectron spectroscopy.^6,12,14^ We have measured the ms-TPES and photoionization spectrum of fulvenone and simulated transitions from the neutral ground X̃^2^A_2_ into both the ground X̃^+2^A_2_ and the first excited Ã^+2^B_1_ cation states in the Franck–Condon approximation.^15^ Fulvenone has, thus, been characterized by different techniques, which helps to trace this crucial intermediate in complex reaction pathways to gain an advanced mechanistic understanding of combustion and lignin valorization processes. However, to obtain reliable kinetics and analytical data, quantification is mandatory. In photoionization measurements, the photoionization cross section (PICS) is an important measure, which relates the mass spectral signal to a concentration in a reaction mixture and can be defined using a reference signal as follows:^16^1
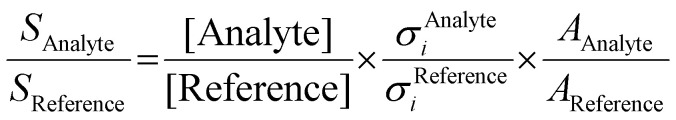
*S*_*n*_ are the photoion signal intensities, [Reference] and [Analyte] are the concentrations of the two species in the reaction mixture, *σ*_*i*_ are the respective ionization cross sections and *A* represents the apparatus function, which accounts for mass-dependent detection efficiencies and flow conditions in the molecular beam expansion.^16,17^ If *A* is unity, eqn (1) can be solved if the *σ*^Reference^_*i*_, [Reference] and [Analyte] are known. Since fulvenone is very reactive, it must be produced *in situ* at a well-known concentration. Due to this complexity, PICSs of reactive species are scarce and they have only been determined for a handful of such species, such as methyl, ethyl, vinyl, propargyl, cyclopropenylidene, allyl, 2-propenyl, phenyl and ethenol.^17–27^ Our strategy to determine the PICS of this important ketene follows the study of Grützmacher and Hübner, who investigated the pyrolysis of salicylic acid, *o*-dinitrobenzene and salicylamide, proposing the last precursor as the most selective and efficient source of fulvenone.^28^

The decomposition of salicylamide 1 is initiated by deamination (R1) yielding the first intermediate, 6-carbonyl-2,4-cyclohexadien-1-one or 2-carbonyl cyclohexadienone (2, C_7_H_4_O_2_), which we call ketoketene 2 from here on. 2 forms fulvenone 3 by sequential decarbonylation (R1). Both CO and NH_3_ are produced together with fulvenone in a 1 : 1 : 1 ratio. The latter can be utilized as reference, because of its well-known PICS and ionization energy close to that of fulvenone 3.^29^
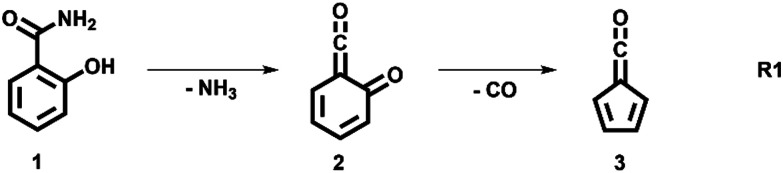


In this contribution, we set out to measure the absolute PICS of the fulvenone ketene utilizing salicylamide as a precursor. We will investigate the salicylamide pyrolysis mechanism to identify possible side reactions and to determine the selectivity of the ketene formation. Therefore, the ms-TPES of the ketoketene 2, the precursor of fulvenone 3, is analyzed in detail, also because of its relevance in the lignin pyrolysis chemistry.^6^ Potential energy surface calculations and computational rate constants will aid the analysis of the experimental findings on salicylamide pyrolysis mechanism to obtain ultimately the PICSs of fulvenone 3 and ketoketene 2.

## Experimental and computational

The experiments were performed using the double imaging photoelectron photoion coincidence endstation (CRF-PEPICO) at the vacuum ultraviolet beamline (VUV) of the Swiss Light Source (SLS) at Paul Scherrer Institute (PSI), Switzerland.^30–32^ Salicylamide (Sigma–Aldrich, 99%) was placed in an in-vacuum sample container (Lenox Laser) with a 100 μm orifice packed by glass wool to prevent clogging. The salicylamide temperature in the sample container was controlled by a copper cube connected to a water thermostat (Huber Minichiller). For experiments requiring high concentration of salicylamide, the water thermostat was replaced by heating cartridges, regulated by a Eurotherm controller. The vaporized salicylamide was mixed with argon (PanGas, 4.8) buffer gas in the sample container and expanded into a *ca.* 35 mm long SiC tubular microreactor (1 mm inner diameter) at 20 sccm mass flow rate. During pyrolysis, the SiC reactor was resistively heated over a length of 15 mm by a DC power supply (Voltcraft) and the temperature was monitored by a Type C thermocouple. The temperature of the gas in the hot reactor could not be determined directly and may be *ca*. 100 K lower than the surface temperature.^33–35^ The reactor is a variant of the Chen-type nozzle, which is operated at an effective pressure of 10–20 mbar and a residence time of 10–50 μs, as pointed out by Guan and Grimm.^33–35^ The gas mixture leaving the microreactor forms a molecular beam upon expansion into high vacuum (10^−5^ mbar), which is skimmed by a 2 mm orifice. The molecules along the centerline of the expansion travel into the ionization chamber (10^−6^ mbar). The gas beam is intersected perpendicularly by synchrotron VUV radiation emitted from a bending magnet. The light is collimated and subsequently dispersed by a monochromator with a 150 mm^−1^ grating providing an *E*/Δ*E* ≈ 1500 energy resolution. The monochromatic VUV radiation is focused onto the exit slit in a differentially pumped rare gas filter (Ar, Ne, Kr) to absorb the higher order radiation. Photoelectrons and -ions are formed in the ionization chamber 50 cm downstream from the focus, accelerated by a constant 213 V cm^−1^ electric field and detected in delayed coincidence by fast delay-line detectors at velocity map imaging (VMI) conditions.^36^ Photoion mass-selected threshold photoelectron spectra (ms-TPES) were plotted by selecting the central, close to zero kinetic energy spot on the electron image, subtracting the hot electron contribution by the procedure of Sztaray *et al.*,^37^ and plotting them in coincidence with ions arriving in the TOF window of interest. Adiabatic ionization energies (AIEs) were corrected for the Stark shift, according to Chupka's expression: Δ*E* = 6.1
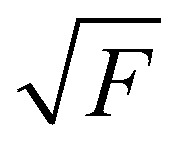
, where Δ*E* is the shift in [cm^−1^] and *F* is the electric field in [V cm^−1^], which results in Δ*E* = 11 meV for a 213 V cm^−1^ field. Photoionization spectra (PIS) were measured by accepting all electrons in coincidence with a photoion of interest as start signal. All spectra were corrected for the photon flux. Quantum chemical calculations were carried out using Gaussian (16 rev. A.03 suite)^38^ and Q-Chem 4.3.^39^ Geometries and unscaled vibrational frequencies of the neutrals and ions were computed at the B3LYP/6-311+G(d,p), CCSD/6-311+G(d,p), MP2/6-311+G(d,p), EOM-IP-CCSD/cc-pVDZ and TD-B3LYP/6-311+G(d,p) levels of theory. Adiabatic ionization energies were calculated using the CBS-QB3, CBS-APNO, G3 and G4 composite methods. Franck–Condon simulations of the ms-TPES were performed using the optimized geometries and vibrational frequencies utilizing Gaussian 16. The stick spectra presented here are at 0 K, while for the convoluted spectrum a temperature of 500 K was assumed for the simulations. Approximate transition state structures were located using constrained geometry scans using B3LYP/6-311++G(d,p) and by synchronous transit-guided quasi-Newton calculations, which were subsequently refined at G4 level of theory to obtain the potential energy surface. The Rice–Ramsperger–Kassel–Marcus (RRKM) theory was applied to calculate the microcanonical rates, using the densities and number of states of the salicylamide ground and transition states, respectively.^40^

## Results and discussion

### Salicylamide pyrolysis

**Fig. 1:**
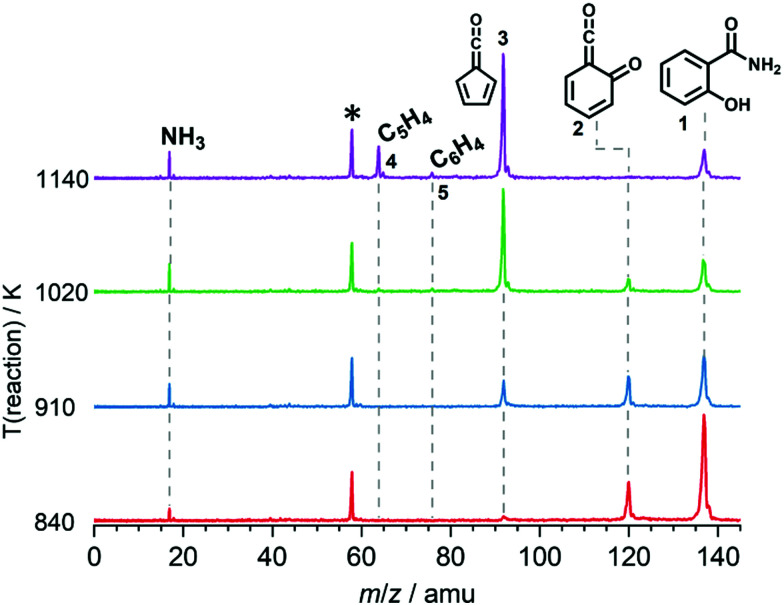
ToF mass spectra of salicylamide pyrolysis recorded at a photon energy of 10.5 eV as function of reactor temperature. Acetone (*m*/*z* 58), an impurity in the chamber, is marked with an asterisk and could be clearly distinguished using ion velocity map imaging.

We explored the salicylamide pyrolysis mechanism in detail by recording temperature-dependent time-of-flight mass spectra (see Fig. 1 and Fig. S1, ESI†). Products and intermediates were isomer-selectively assigned based on their photoion mass-selected threshold photoelectron (ms-TPE) spectrum, as compared with Franck–Condon simulations (Fig. S2, ESI†) and accurate ionization energy calculations (Table S1, ESI†), or with reference spectra. As shown in Fig. 1, the ketoketene **2** (*m*/*z* 120), fulvenone **3** (*m*/*z* 92) and C_5_H_4_ isomers **4** (*m*/*z* 64), mainly penta-1,2-dien-4-yne, are formed. In addition, C_6_H_4_ isomers **5** (*m*/*z* 76), such as benzyne and hexadiynes, and ammonia (*m*/*z* 17) are produced upon pyrolysis of salicylamide.

Upon increasing the reactor temperature, the salicylamide 1 (*m*/*z* 137) and ketoketene 2 peaks decreased while the fulvenone 3 and NH_3_ signals increased. Between 1020 and 1140 K, fulvenone dominates the mass spectrum at 10.5 eV. Except for CO, no side products were observed. As CO is ionized above 14 eV, it is not seen in Fig. 1. As reported by Ormond *et al.*,^6^ ketoketene 2 forms fulvenone 3 by decarbonylation and subsequent ring contraction. Therefore, 2 must be fully converted into 3 for the PICS measurement, since only then are ammonia and fulvenone produced in a known, 1 : 1 ratio, required for ammonia to be used as reference in eqn (1). At 1020 K reactor temperature, 2 showed only a small peak at *m*/*z* 120, which disappears completely once the temperature is increased to 1140 K. The C_5_H_4_4 and C_6_H_4_ isomers’ 5 peaks, however, rose simultaneously at this temperature. Note that C_5_H_4_ isomers originate from the decarbonylation of fulvenone 3 (R2)^6^ while C_6_H_4_ isomers were yielded by CO_2_ loss from 6-carbonyl-2,4-cyclohexadien-1-one 2 in (R3):
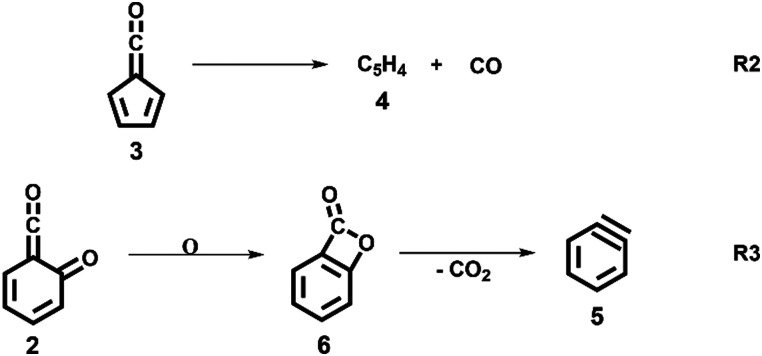


Thus, different reaction temperatures between 1020 and 1140 K were thoroughly investigated and the signals of *m*/*z* 64, 76, 92 and 120 were integrated and presented in Fig. S3 (ESI†). We also noticed that dissociative ionization of ketoketene 2 (*m*/*z* 120) yields fulvenone cations (*m*/*z* 92), too, as shown in Fig. S4 (ESI†). By increasing the reactor temperature, the concentration of salicylamide 1 in the molecular beam was lowered and the dissociative ionization contribution to fulvenone was minimized. Therefore, the photoionization spectrum (PIS) of fulvenone for the PICS measurement (see below) was recorded at 1050 K reactor temperature and 338 K sample container temperature (determined by the thermocouple outside of sample container) at a selectivity of 87%. Still, the formation of C_6_H_4_ isomers (*m*/*z* 64), such as benzyne, could not be fully supressed and needs to be further investigated, as it is in direct competition with the fulvenone 3 generation from the ketoketene 2. In fact, ketoketene was also observed during the pyrolysis of lignin model compounds, such as salicylaldehyde, and may play an important role in this process.^6^ Thus, in the next chapter we focus on the spectroscopic characterization of the ketoketene (2, *m*/*z* 120) and its fate at higher pyrolysis temperature to yield 3 and 5.^6^

### Threshold photoelectron spectrum of ketoketene 2

**Fig. 2:**
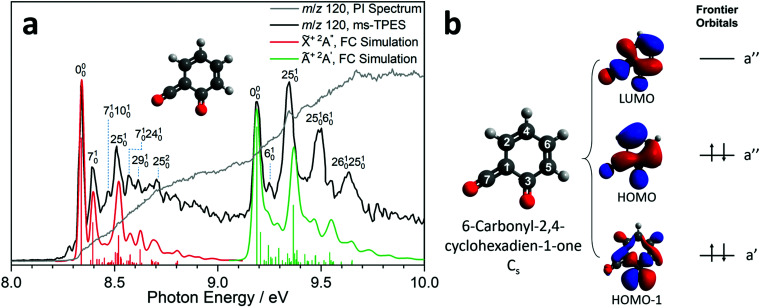
(a) ms-TPES (black trace) and PI spectrum (grey trace) of 6-carbonyl-2,4-cyclohexadien-1-one together with Franck–Condon (FC) simulations for the X̃^+ 2^A′′ ← X̃^1^A′ (red trace) and Ã^+2^A′ ← X̃^1^A′ (green trace) transitions. The harmonic frequencies and geometries of neutral and ion are calculated at B3LYP/6-311+G(d,p) level of theory. (b) Energy diagram of the three frontier orbitals of 6-carbonyl-2,4-cyclohexadien-1-one calculated at the B3LYP/6-311+G(d,p) level of theory.

Due to the elusive nature of ketoketene 2, studies on this intermediate are few and far between. In 1979, Schulz and Schweig measured the photoelectron spectrum in the 8–18 eV energy range and assigned the ionization bands.^11^ Chapman *et al.* measured the IR spectrum of the ketoketene 2 in an Ar matrix at 8 K and identified its characteristic bands at 2139 and 1650 cm^−1^. In addition, they found contributions of benzpropiolactone, which could be reversibly converted to ketoketene 2 by UV irradiation. However, prolonged irradiation of 2 produced benzyne and CO_2_.^41^ The reactivity of 2 and the formation of 3 was also investigated by Wentrup *et al.* in matrix isolation.^42^ In this section, we investigate the photoion mass-selected threshold photoelectron spectrum (ms-TPES) of 2 between 8–10 eV with 5 meV resolution. Aside from characterizing ketoketene, the results will also be used to underpin the ketoketene decomposition mechanism to fulvenone 3 and to understand the side reactions. Fig. 2a shows the ms-TPES of ketoketene 2 obtained by salicylamide 1 pyrolysis at *ca.* 850 K in the SiC reactor. The TPE signal begins to rise at about 8.21 eV and shows three vibrational bands at 8.34, 8.39 and 8.51 eV, after which the signal declines at 8.7 eV. The adiabatic ionization energy (AIE) is determined to be 8.35 ± 0.01 eV (Table 1) considering the Stark-shift (11 meV).^43^ In conventional PES literature, Schulz and Schweig assigned the first band to the X̃^+2^A′′←X̃^1^A′ transition and reported an ionization energy of 8.43 eV.^11^ The small band at *ca.* 8.29 eV, as well as the width of the transitions are due to hot and sequence band transitions induced by insufficient cooling of the molecular beam after exiting the hot pyrolysis reactor.^44^ With increasing photon energy, the TPE signal increases again at around 9.13 eV and exhibits five resolved bands at 9.19, 9.25, 9.34, 9.49 and 9.64 eV. This set of bands agree well with the Ã^+2^A′ ← X̃^1^A′ transition assignment of Schulz and Schweig (IE = 9.38 eV).^11^

**Table 1:** Summary of the experimentally and theoretically obtained ionization energies of 6-carbonyl-2,4-cyclohexadien-1-one

Method	(X̃^+2^A′′)	(Ã^+2^A′)
	Adiabatic ionization energy (AIE)/eV	
Experiment:		
ms-TPES	8.35	9.19
Theory:		
B3LYP/6-311+G(d,p)	8.27	9.02
CBS-QB3	8.30	—
CBS-APNO	8.29	—
G3	8.42	—
G4	8.36	—
CCSD/6-311+G(d,p)	8.01	—
MP2/6-311+G(d,p)	9.03	—
ms-TPES (band max.)	8.35	9.34
PES^11^ (band max.)	8.43	9.38
EOM-IP-CCSD/cc-pVTZ (VIE)	8.45	9.53

Furthermore, the EOM-IP-CCSD/cc-pVTZ calculated vertical ionization energy (VIE) of 9.53 eV (see Table 1) verifies our assignment for the transitions between 9.19 and 9.64 eV computationally. Besides the ms-TPES, the PIS is shown in Fig. 2a. It exhibits a step-like onset close to the first ionization energy and linearly increases afterwards but exhibits plateaus in the 8.8–9.1 eV and 9.7–10.0 eV photon energy ranges. The constant PI signal between 8.8–9.1 eV is mirrored by the slightly decreasing ms-TPES, indicative for dropping Franck–Condon factors. To further understand the electronic structure, we carried out B3LYP/6-311+G(d,p) calculations on 2. Fig. 2b shows the three frontier orbitals of the *C*_s_ symmetric neutral ketene (X̃^1^A′) and Table S2 (ESI†) summarizes the most important geometry changes upon photoionization. According to Koopmans' theorem, removing an electron from the highest occupied molecular orbital (HOMO) yields the X̃^+2^A′′ cation state. The HOMO has bonding components along the C2=C4 double bond as well as at the C6=C5 position. At the carbonyl C3=O and ketene function C7=O, the HOMO possesses antibonding character. Once the ketoketene 2 is ionized, the electron density decreases, leading to an increase of bond lengths (C2=C4, C6=C5, and C1=C7), as an electron is removed from a formally binding orbital component. In contrast, the C1–C2 and C4–C6 bond lengths are shortened, because the antibonding character of these bonds decreases. A similar pattern was also found in fulvenone 2 ionization.^15^ In general, the bond angles are only slightly affected upon ionization into the ground state cation.

ms-TPE spectroscopy is a unique tool to detect reactive intermediates and to selectively identify isomers in harsh environments.^45,46^ Its full potential can only be realized if both the electronic structure and the vibrational features in the ms-TPE spectrum are fully understood. The latter can be accurately simulated by applying the Franck–Condon principle in the double harmonic approximation at the optimized geometries of neutral and cation state, while also including the Duschinsky rotation.^47^ The FC predicted spectrum of the X̃^+2^A′′ (red trace) and Ã^+2^A′ (green trace) cation states of ketoketene 2 are depicted in Fig. 2a. The 0–0 transition to the X̃^+2^A′′ cation state is located at 8.35 eV, followed by excitations in several vibrational modes active upon photoionization. The lowest transition energy is 50 meV (403 cm^−1^) above the origin and is assigned to the *v*_7_ ring deformation mode at a theoretical value of 451 cm^−1^ at the B3LYP/6-311+G(d,p) level of theory. At 8.47 eV, the experimental transition energy is 1371 cm^−1^ which compares well to the theoretical value of 1456 cm^−1^ (*v*_25_) of a ring deformation mode. The third band with a shift of 0.27 eV (2177 cm^−1^) with respect to the origin is a C=O stretching vibration with a computed transition energy of *v*_29_ = 2296 cm^−1^. Furthermore, we observe combination bands of *v*_7_ and *v*_10_ and *v*_24_, the latter two are assigned to further deformation ring modes.

**Fig. 3:**
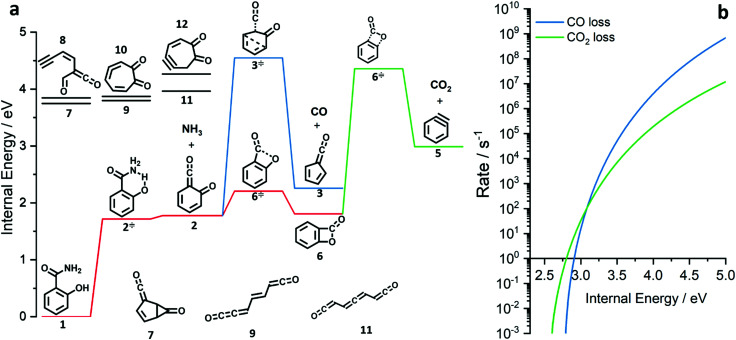
Potential energy surface of the salicylamide pyrolysis (a) and comparison of RRKM rate constant of the CO and CO_2_ loss reactions (b).

The active vibrations mirror the geometry change upon ionization as elicited by the removal of a HOMO electron, as discussed above. Schulz and Schweig assigned the band above 8.8 eV to the excited state of 6-carbonyl-2,4-cyclohexadien-1-one cation at a IE of 9.38 eV,^11^ which agrees with the most-prominent band at 9.34 eV in our ms-TPES. Our computational results for the Ã^+2^A′ state of the keteoketene 2 cation, which finds a VIE at 9.53 eV at EOM-IP-CCSD/cc-pVTZ level of theory (see Table 1), also confirms this assignment. While the band at 9.19 eV is the origin transition into the Ã^+2^A′ state, the band at 9.25 eV is assigned to the *v*_6_ mode at a transition energy of 450 cm^−1^ at B3LYP/6-311+G(d,p) level of theory. This stretching vibration at C1–C3 and C4–C6 agrees with the change of the bond angles at C2=C4–C6 and C4–C6=C5 upon transition into the Ã^+2^A′ state and is expected from the removal of an electron from the HOMO−1. Besides the *v*_6_ vibration, the *v*_25_ mode (1422 cm^−1^) is active and shows a ring deformation mode dominated by the stretching of C1–C3 and C3=O bonds, thereby leading to a significant change in C6=C5–C3 and C5–C3–C1 angles as well as the bond length in C1–C3, C1=C7, C3–C5 and C3=O (see Table S2, ESI†). Above 9.4 eV, there are at least two further vibrational features assigned to combination bands of the mentioned transitions. The FC simulation of the Ã^+2^A′ state shows good agreement with the experimental spectrum below 9.45 eV. However, the intensity of the second band at 9.34 eV is not reproduced well. EOM-IP-CCSD/cc-pVDZ calculations for the X̃^+2^A′′ and Ã^+2^A′ states confirmed the harmonic DFT/TDDFT FC factors. We also investigated other isomers which may contribute to the ms-TPES above 9.2 eV, which are depicted in Fig. 3 and Fig. S5 (ESI†). From the seven computationally investigated isomers, only benzpropiolactone (7-oxabicyclo[4.2.0]octa-1(6),2,4-triene-8-one) 6 is almost isoenergetic to the ketoketene 2, while the other isomers (7–12, see Fig. 3) are more than 2 eV less stable and may require much higher barriers to be formed. In addition, isomers 7–12 are likely to decompose to C_2_ or C_3_ carbon chains at higher pyrolysis temperatures, which were not observed experimentally. Furthermore, the calculated FC envelopes and ionization energies of isomers 7–12 do not match to the features observed in the ms-TPES of *m*/*z* 120 (see Fig. S5, ESI†). Yet, when investigating the ionization of benzpropiolactone 6 computationally, we found that the C–O bond of the 4-membered ring opens upon ionization to form the ketoketene 2^+^ cation. This large geometry change leads to unfavourable FC factors at the calculated adiabatic ionization energy of 8.31 eV (G4). The EOM-IP-CCSD/cc-pVTZ computed VIE, on the other hand, is located at 9.19 eV. Thus the differences between the experimental spectrum and the FC simulations (Fig. 2) may perhaps be explained by intensity borrowing from isomer 6, which was also observed in the matrix IR studies by Chapman *et al.*^41,48^

### Salicylamide pyrolysis mechanism

The ms-TPES data and the observation of benzyne 5 points to contributions from a second *m*/*z* 120 isomer, and that the salicylamide pyrolysis mechanism cannot solely explained by R1. A thorough description of the mechanism and the side reactions is essential to obtain trustworthy ionization cross sections of fulvenone 3. Here, we focus on the formation of *m*/*z* 120 isomers 2 and 6, and their decomposition reactions leading to fulvenone 3 and benzyne 5. The potential energy surface of salicylamide pyrolysis was calculated as shown in Fig. 3a. Upon hydrogen migration from the OH group to the amide group in 1, a deamination is initiated, which yields the ketoketene 2 at 1.75 eV (G4) after passing a submerged barrier of 1.71 eV (
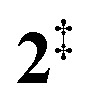
, G4).^49^ Based on our experimental results, the keteoketene 2 will dominantly lose CO and ring contract to form fulvenone 3 (R1), which involves overcoming a barrier at *ca.* 2.77 eV (
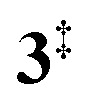
). Interestingly, the keteoketene 2 can ring-close to the almost isoenergetic benzpropiolactone 6, at a low barrier of only 0.43 eV (2 → 
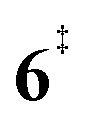
 → 6). This is in line with the IR matrix study,^41,48^ where isomers 2 and 6 were shown to be present simultaneously and may also explain the deviations of the experimental *m*/*z* 120 ms-TPE spectrum from the FC-simulated one in Fig. 2a. A subsequent decarboxylation of 6 yields benzyne 5 by overcoming a transition state at a relative high barrier of 2.58 eV (
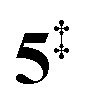
). Yet, this barrier is lower than 
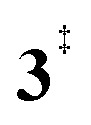
 leading to fulvenone, which seems to suggest that benzyne should be the dominant product, in contrast to the observed dominance of fulvenone 2, as seen in Fig. 1. Microcanonical rate constants were calculated and are presented in Fig. 3b comparing CO and CO_2_ loss over the respective transition states, based on the Rice–Ramsperger–Kassel–Marcus (RRKM) theory. They indicate that decarboxylation (6 → 
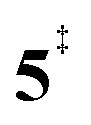
 → 5) only dominates below an internal energy of *ca.* 3 eV. In this energy range, rates are slow compared with the *ca.* 100 μs residence time in the microreactor and unimolecular fragmentation will not take place prior to detection. Decarbonylation (2 → 
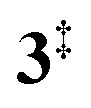
 → 3) takes over at higher internal energies, which are expected to lead to product formation in the microreactor. At internal energies as high as 4.5 eV, decarbonylation is almost two orders of magnitude faster than decarboxylation. This is in agreement with the experimental product distribution of close to 90% fulvenone and benzyne never exceeding 4% (see Fig. S3, ESI†). Based on this decomposition mechanism, we can now determine the PICS of both fulvenone 3 and the ketoketene 2 intermediates.

### Photoionization cross section of fulvenone and ketoketene

Salicylamide produces primarily ketoketene 2 together with ammonia at lower temperatures. Ketoketene 2 is fully converted to fulvenone 3 and CO at higher reactor temperatures, while the ammonia signal remains constant. Thus, ammonia is directly related to fulvenone 3 and the ketoketene 2 as both are produced in a 1 : 1 ratio and can therefore be used as calibrant for the PICS. Here we rely on the PICS of ammonia determined by Xia *et al.*, which has an estimated error less than ± 10% when compared to the Watanabe *et al.* results.^29,50,51^ Furthermore, the fulvenone and ammonia parent ions are stable below a photon energy of 10.5 eV and do not undergo fragmentation. Thus, the PICS can be determined using eqn (2):2
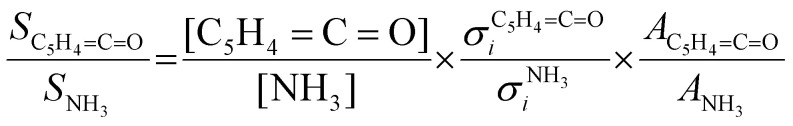
*S*_*n*_ is the detected ion signal of the fulvenone and NH_3_, while [C_5_H_4_=C=O] and [NH_3_] correspond to the relative concentrations of fulvenone and ammonia in the molecular beam. *σ*^*n*^_*i*_ are the PICS of fulvenone and NH_3_, while *A* represents the apparatus function. The latter is the mass-discrimination factor, which is attributed to mass-dependent detection probability and flow conditions in the molecular beam expansion (see ESI†) and was confirmed to be unity by measuring various gas mixtures (Fig. S6, ESI†). Since ammonia and fulvenone are produced in a 1 : 1 ratio, eqn (2) can be simplified as follows:3
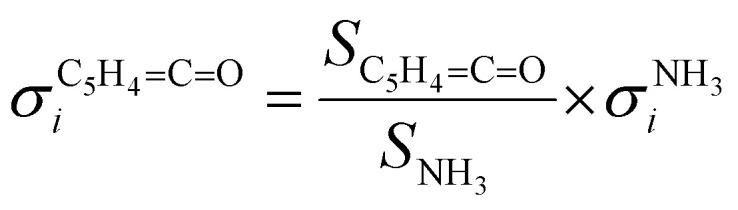


**Fig. 4:**
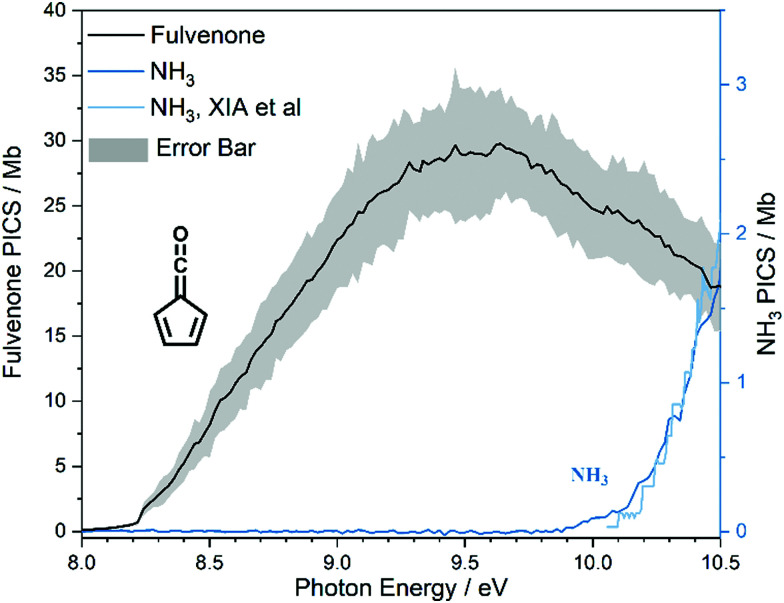
The ionization cross section of fulvenone shown with a 20% range of uncertainty.

The photoion signal of ammonia was normalized to the PICS 
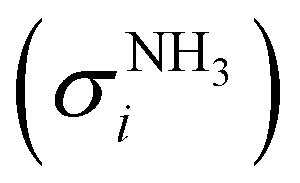
 from Xia *et al.*^29^ and the fulvenone PIS was corrected with the same factor to yield the PICS (see Fig. 4 and Table S3, ESI†). By applying this procedure we obtain a PICS of ammonia from our PI data of 0.88 Mb at 10.35 eV, which is within the 10% error bar from the literature.^29,50,51^ Due to the nature of the pyrolysis source, reactive species are produced vibrationally hot, which leads to the appearance of hot and sequence bands below the adiabatic ionization energy of 8.25 eV.^15^ Above the IE, the PICS monotonically increases until a plateau region between 9.3 and 9.6 eV is reached, while it decreases afterwards. In the following, we discuss the error bars of our fulvenone PICS measurement. The mass discrimination in the effusive molecular beam is close to unity, and integration effects, such as the ^13^C isotopologue, as well as subtraction of the false coincidence signal and the photon flux correction are considered minor errors, which account for less than 5% of the absolute error. At the best measurement conditions for 3, we found about 6% unconverted ketoketene (2, at *m*/*z* 120), while the decarboxylation product benzyne accounts for up to 2% of the total ion signal (see Fig. S3, ESI†).

In addition, fulvenone decomposes to C_5_H_4_ species (see Fig. S2, ESI†), which contributes further 5% to the uncertainty, yielding a total of *ca.* 11% by error propagation. Considering the temperature dependence of the ion signal due to the contribution of hot and sequence bands, reproducibility effects and the uncertainty in the cross section measurements of ammonia (10%),^29,50,51^ leads us to a total error bar of up to 20%. The latter is indicated as grey shaded area in Fig. 4 and agrees with the typical error bar for PICS measurements in the literature.^16,20^

**Fig. 5:**
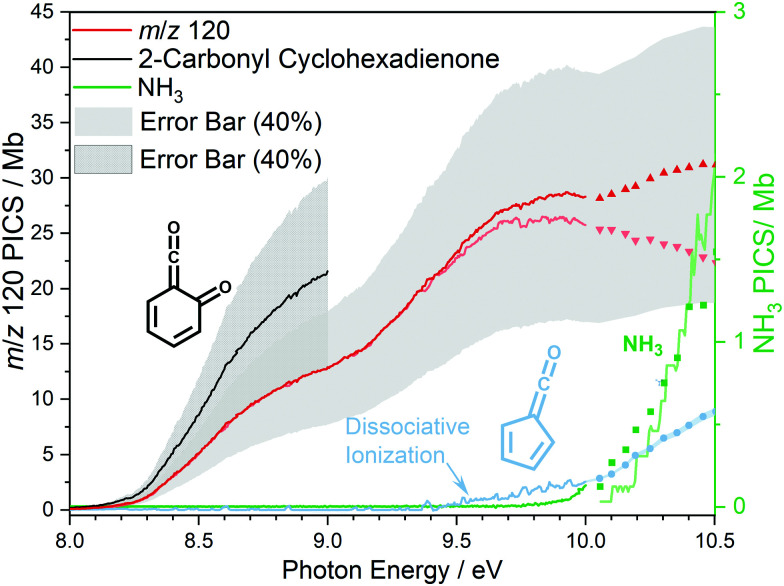
The lumped photoionization cross section (red curve) of the *m*/*z* 120 species 2-carbonyl cyclohexadienone 2 and benzpropiolactone 6 along with the fulvenone fragment (blue curve). Effective PICS of the ketoketene 2 is depicted in black. We assume a conservative error bar of 40%.

At 840 K reactor temperature, fulvenone (*m*/*z* 92) formation does not yet play a role and the ammonia signal can be used to determine the PICS of the ketoketene 2 species at *m/z* 120, which is depicted in Fig. 5 (red curve). Several assumptions had to be taken into account to determine this physical quantity: Dissociative ionization of the ketoketene 2 in a decarbonylation reaction above 9.5 eV produces *m*/*z* 92. The corresponding ion signal is confirmed to be due to dissociative ionization by velocity map ion images (VMIs), which are sensitive to kinetic energy release, as depicted in Fig. S4 (ESI†). Since the DPI onset of *m*/*z* 120 to *m*/*z* 92 is similar to the ionization energy of the calibrant, ammonia, both species (*m*/*z* 120 and 92) were added together to get the total PICS (red curve Fig. 5). In addition, fulvenone is also produced *via* pyrolysis, and was subtracted from the *m*/*z* 92 signal, utilizing the fulvenone PI curve at full conversion (Fig. 4), yielding solely the DPI signal (see Fig. 5, blue trace). It is intriguing to compare now the PICS of fulvenone 3 (Fig. 4) with the one of *m/z* 120 in Fig. 5 (red curve), which is about a factor of two lower at 9 eV. This may be owing to contributions of the bicyclic benzpropiolactone 6 isomer, which may account for 40% of the 120 amu population in the reaction mixture at 840 K pyrolysis temperature, due to it being only 30 meV less stable than 2 (Fig. 3 a) and the presence of a low-lying isomerization transition state 
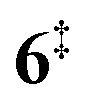
. Owing to the large geometry change upon ionization, as discussed in the TPES section (AIE = 8.34 eV *vs.* VIE = 9.19 eV) the FC factors of isomer 6 are likely negligible below 8.9–9.0 eV photon energy. Thus, only ketoketene 2 contributes significantly to the PICS below 9 eV. However, the reference ammonia is formed in a 1 : 1 ratio to the analytes 2 + 6, which must be considered in eqn (1) to derive the cross section for 2 in the energy range where 6 is not expected to contribute. By scaling the PICS signal up by 1.67, according to relative abundance of 2 in 2 + 6 at 840 K, we derive an effective PICS for the ketoketene 2 at low photon energies (black curve in Fig. 5). Due to these considerations, we increased the error bars of the *m*/*z* 120 PICS measurements conservatively to about 40%, also including the discussed errors from the fulvenone measurements. We would like to point out that in all hot reactive environments (combustion & catalysis) both isomers 2 and 6 are likely to be present due to their isoenergeticity and a separation may not be possible at higher photon energies. Our experimental PICS data at *m/z* 120 provides a lumped PICS of 2 and 6 (red curve in Fig. 5) above 9 eV, which may still enable a semi-quantitative analysis of both isomers in reaction mixtures.

## Summary and conclusion

Fulvenone was produced in salicylamide pyrolysis together with ammonia and CO to determine its photoionization cross section. By investigating the reaction mixture using PEPICO detection to plot the photoion mass selected TPES, we could prove that salicylamide is converted to 6-carbonyl-2,4-cyclohexadien-1-one (ketoketene, 2) in a deamination reaction after hydrogen transfer to the NH_2_ group from the vicinal OH group. In the ms-TPE spectrum of 2, the first band is assigned to the X̃^+2^A′′ state with an AIE of 8.35 eV, in excellent agreement with composite method calculations. The vibrational transitions are mainly ring deformation and C=C stretching modes according to FC simulations and agree well with the geometric changes upon ionization and molecular orbital considerations. The second band at 9.18 eV is assigned to the Ã^+2^A′ state, and the vibrational progression of the band is dominated by the C3=O stretching vibration. The FC simulation agrees well with the experimental spectrum at the onset of the band, but the agreement breaks down at higher energies. This may be owing to intensity borrowing from and contributions of the bicyclic ketoketene isomer benzpropiolactone 6.

Upon increasing the temperature, ketoketene 2 decarbonylates to yield fulvenone ketene 3. We have found a second decomposition channel of the ketoketene 2, which leads to the benzyne 5 side product by decarboxylation. Potential energy surface calculations starting from salicylamide show that deamination proceeds over a barrier of less than 1.8 eV to yield the ketoketene 2. Thereafter, parallel CO_2_ or CO loss, yields benzyne 5 or fulvenone 3, respectively. Although the mass spectra suggest that fulvenone is the dominant product, the barrier to CO_2_ loss is lower than that to CO loss. Nevertheless, RRKM rate constant calculations show that the decarbonylation over a looser transition state outcompetes decarboxylation by more than one order of magnitude in the energy range where rates become commensurate with the residence time in the microreactor and in agreement with experimental observations. Equipped with this knowledge, we could determine a reliable photon-energy-dependent ionization cross section of fulvenone ketene 3 for the first time. Corresponding to different measurement uncertainties, a typical error bar of 20% was estimated. At a typical laser photon energy of 10.48 eV (3 × 355 nm) the PICS of fulvenone 3 was found to be 18.8 ± 3.8 Mb. The cross section of the ketoketene 2 could also be obtained at lower pyrolysis temperatures. However, due to non-suppressible side reactions such as dissociative ionization of the ketoketene 2, fulvenone 3 and benzpropiolactone 6 formation, we had to assume more conservative error bars for *m*/*z* 120. Nonetheless, the PICS of 2-carbonyl cyclohexadienone (ketoketene) could be estimated below the VIE of 6, by considering the thermal equilibrium between isomers 2 and 6. We have determined an effective photoionization cross section of 2 of 21.5 Mb at 9 eV, which compares with 21.9 Mb of fulvenone PICS.

Our PICS measurements enable the quantification of highly reactive ketenes in lignin valorization and combustion processes using photoionization mass spectrometric tools. This will make kinetics data accessible to determine the relative contribution of parallel reaction pathways in the lignin catalytic pyrolysis reaction mechanism, which will in turn aid the bottom up optimization of this process.^46^

## Author contributions

ZP: investigation, visualization, validation, formal analysis, writing manuscript. AB: discussion of the data, corrections to the manuscript. JAvB: discussion of the data, corrections to the manuscript, thesis supervisor. PH: conceptualization, investigation, validation, writing manuscript, supervision, project administration, funding acquisition.

## Conflicts of interest

There are no conflicts to declare.

## Supplementary Material

CP-024-D1CP05206C-s001
