# Supplementary material for: On the absolute photoionization cross section and threshold photoelectron spectrum of two reactive ketenes in lignin valorization: fulvenone and 2-carbonyl cyclohexadienone
Source: Phys Chem Chem Phys. 2022 Jan 26;24(6):3655–63. doi: 10.1039/d1cp05206c (PMC8827046; doi:10.1039/d1cp05206c)
Supplement: CP-024-D1CP05206C-s001 [file CP-024-D1CP05206C-s001.pdf]

This journal is © the Owner Societies 2022

ELECTRONIC SUPPLEMENTARY INFORMATION

On the Absolute Photoionization Cross Section and Threshold Photoelectron Spectrum of two Reactive Ketenes in Lignin Valorization: Fulvenone and 2-Carbonyl Cyclohexadienone

Zeyou Pan[a,b], Andras Bodi[a], Jeroen A. van Bokhoven[a,b], Patrick Hemberger [a]*

[a] Zeyou Pan, Andras Bodi, Jeroen A. van Bokhoven and Patrick Hemberger,
Paul Scherrer Institute, 5232 Villigen, Switzerland. E-mail: patrick.hemberger@psi.ch.
[b] Zeyou Pan and Jeroen A. van Bokhoven
Institute for Chemical and Bioengineering, Department of Chemistry and Applied Biosciences, ETH Zurich, 8093 Zurich, Switzerland

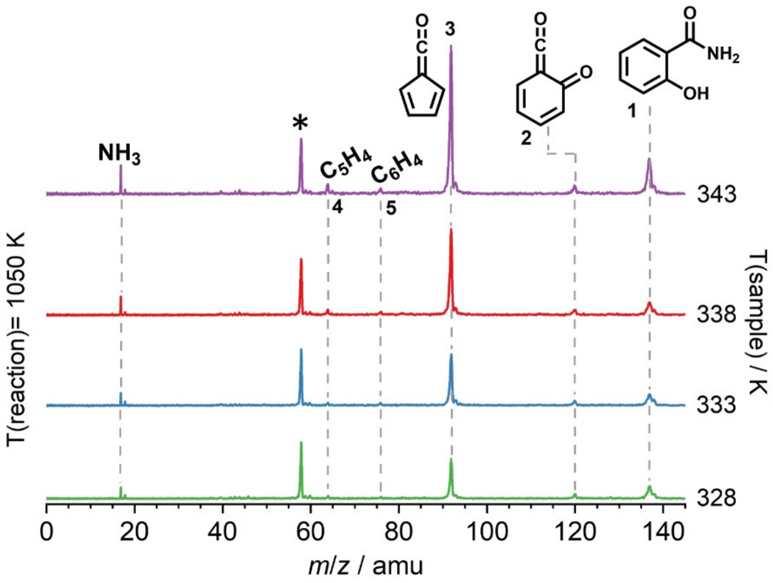

Figure S1. ToF mass spectra of salicylamide pyrolysis as a function of sample temperature, i.e., sample partial pressure in the reactive mixture. The sample temperature was read by the thermocouple on the outside of sample container. *: acetone, an impurity in the chamber. The effect of sample concentration on the pyrolysis products was investigated at a reactor temperature of 1050 K. By decreasing the sample temperature starting from 343 K, the signals of ketoketene **2**, C_6H_4 and C_5H_4 isomers decreased. At 328 K, the signals of C_6H_4 and C_5H_4 isomers as well as the ketoketene **2** were barely visible however, fulvenone **3** signal went down dramatically.

Table S1. Ionization energy of benzenediol products.

Compound	*m/z*	IE (eV)		Ref. IE (eV)	Reference
		Calculated[a]	Experimental[b]		
Ammonia	17	10.164	10.180	10.2	[1]
Penta-1,2-dien-4-yne	64	9.234	9.215	-	-
Penta-1,3-diyne	64	9.505	9.485	9.50 ± 0.02	[2]
Penta-1,4-diyne	64	10.313	10.282	10.27 ± 0.02	[3]
1,2,3,4-Pentatetraene	64	8.772	8.768	8.67	[4]
Benzyne	76	9.605	9.725	9.64	[5]
Hexa-1,2,3,4,5-pentaene	76	8.497	8.384	-	-
(Z)-Hex-3-en-1,5-diyne	76	9.105	9.092	9.10 ± 0.02	[3]
(E)-Hex-3-en-1,5-diyne	76	9.078	8.974	9.07 ± 0.02	[3]
Fulvenone	92	8.244	8.271	8.05	[6]
6-Carbonyl-2,4-cyclohexadien-1-one	120	8.357	8.341	8.43 (vertical value)	[7]

a: Adiabatic ionization energies (AIE) are calculated at the G4 level of the theory.

b: The value from the first vibrational peak.

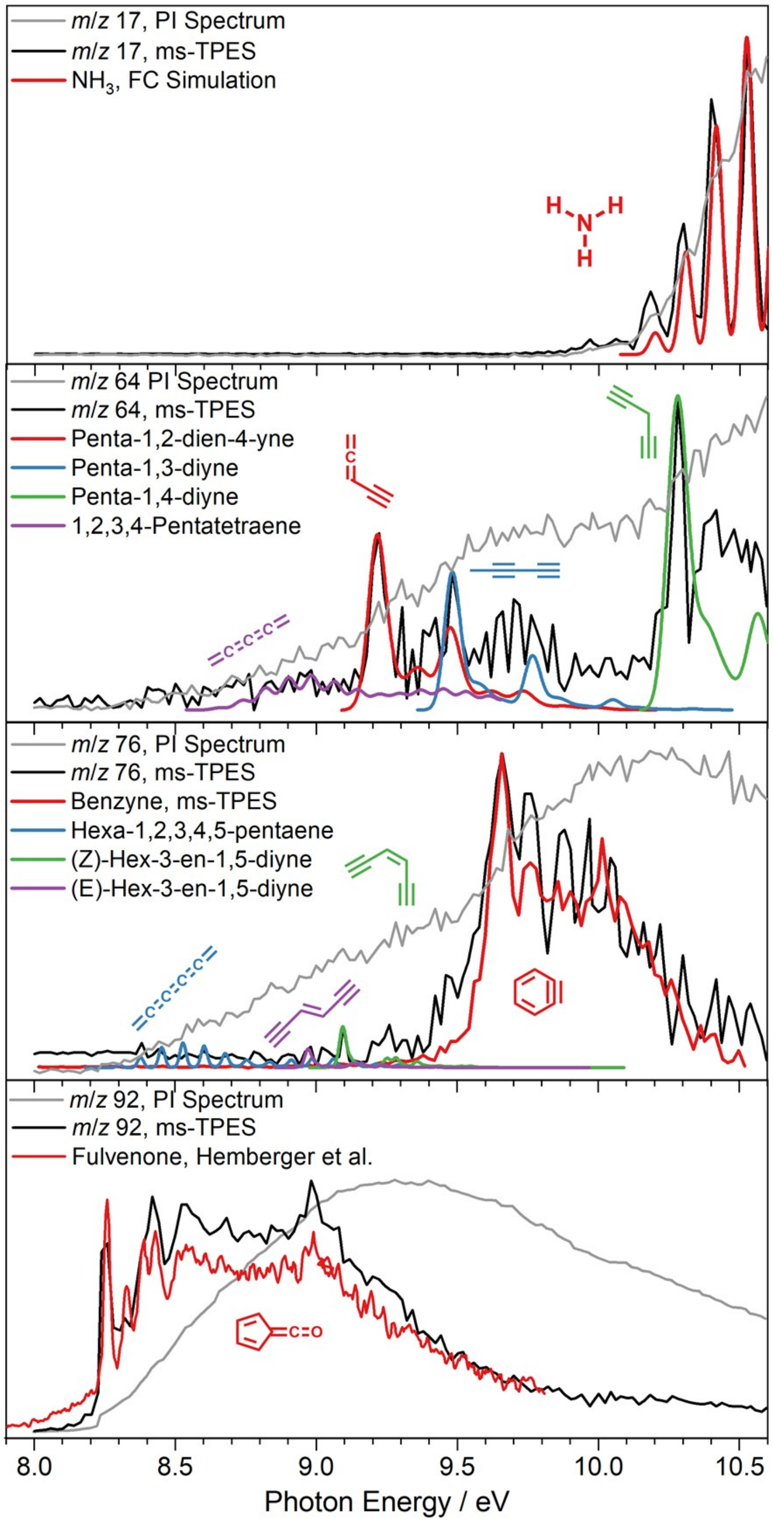

Figure S2. Photoion mass-selected threshold photoelectron (ms-TPE) and photoionization spectra upon salicylamide pyrolysis shown together with Franck–Condon (FC) simulations or reference spectra.[8]

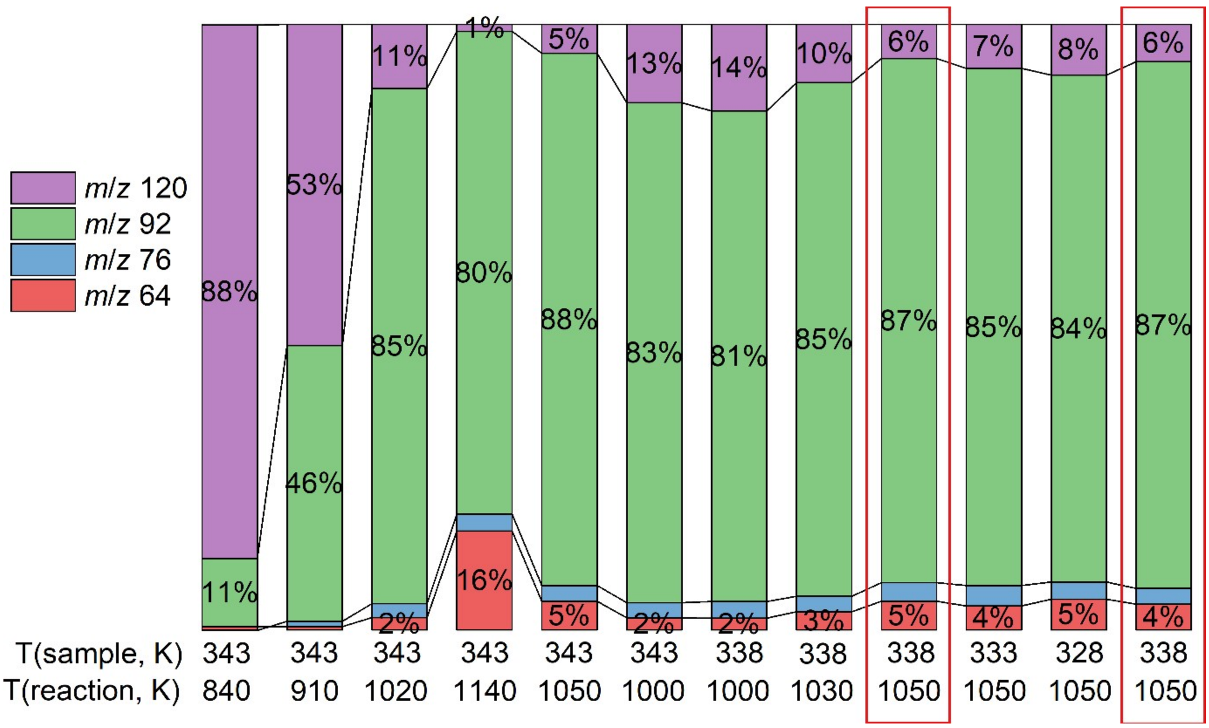

Figure S3. The product distribution of *m/z* 64, 76, 92 and 120 in percentage with various reaction conditions (sample temperature and reaction temperature).

Table S2. 6-Carbonyl-2,4-cyclohexadien-1-one geometries of $\tilde{X}$ $^1A_1$, $\tilde{X}^+$ $^2A''$ and $\tilde{A}^+$ $^2A'$ state different level of theory. Bond lengths are given in Å, bond angles in degrees.

Level of Theory	$\tilde{X}$ $^1A'$	$\tilde{X}^+$ $^2A''$	$\tilde{X}$ $^1A'$	$\tilde{X}^+$ $^2A''$	$\tilde{A}^+$ $^2A'$
	CCSD/cc-pVDZ	EOM-IP-CCSD/cc-pVDZ	B3LYP/6-311+G(d,p)		
$R(C_1–C_2)$	1.461	1.403	1.44471	1.39580	1.43671
$R(C_1–C_3)$	1.493	1.489	1.49975	1.49359	1.44383
$R(C_1=C_7)$	1.357	1.407	1.33954	1.37846	1.36578
$R(C_2=C_4)$	1.363	1.409	1.35551	1.40488	1.36508
$R(C_3–C_5)$	1.480	1.463	1.45825	1.45416	1.42485
$R(C_4–C_6)$	1.462	1.428	1.43829	1.40668	1.41797
$R(C_5=C_6)$	1.366	1.385	1.35862	1.38238	1.37304
$R(C_2–H)$	1.095	1.095	1.08339	1.08406	1.08311
$R(C_4–H)$	1.094	1.095	1.08256	1.08333	1.08169
$R(C_5–H)$	1.095	1.095	1.08355	1.08421	1.08132
$R(C_6–H)$	1.097	1.095	1.08537	1.08396	1.08496
$R(C_3=O)$	1.228	1.234	1.22719	1.22702	1.27269
$R(C_7=O)$	1.154	1.131	1.14673	1.12635	1.13036
$\alpha(C_1–C_2=C_4)$	118.9	117.6	119.20649	118.84176	119.48284
$\alpha(C_1=C_7=O)$	179.2	178.8	178.79415	178.66755	178.89383
$\alpha(C_2=C_4–C_6)$	120.0	121.4	120.11478	121.29303	119.67188
$\alpha(C_4–C_6=C_5)$	122.6	121.7	122.68228	121.28348	123.13400
$\alpha(C_6=C_5–C_3)$	122.0	120.7	122.33054	121.41923	119.09933
$\alpha(C_5–C_3–C_1)$	113.2	114.9	113.06811	114.89203	118.12458
$\alpha(C_3–C_1–C_2)$	123.1	123.8	122.59780	122.27048	120.48737

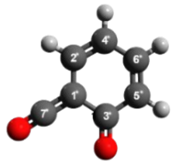

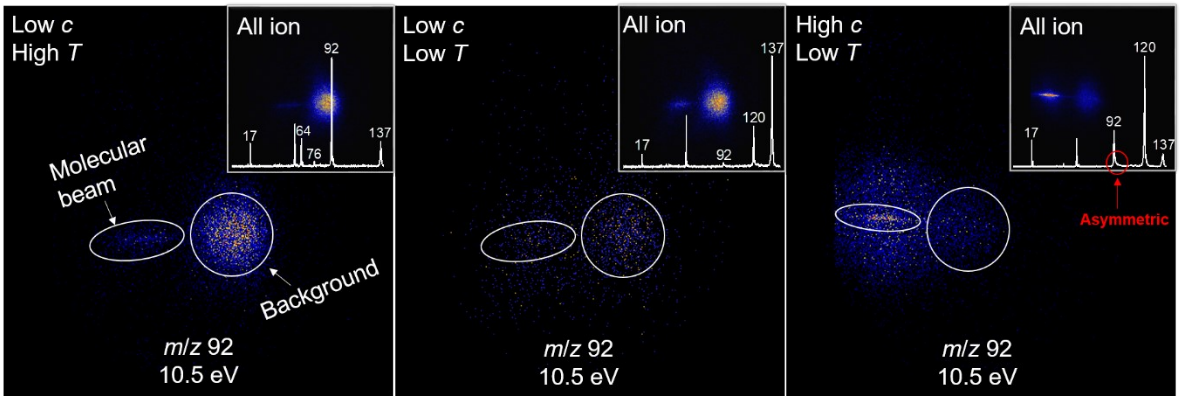

Figure S4. Velocity map ion images measured during the pyrolysis of salicylamide. *c*: sample concentration; *T*: reaction temperature. The left image shows the photoionization of fulvenone (*m*/*z* 92) at high temperature and low concentration in the molecular beam. Two features are apparent: A narrow velocity distribution of the molecular beam component perpendicular to the propagation axis and a broad room temperature background that stems from rethermalized fulvenone scattered in the chamber. The mass spectrum of salicylamide pyrolysis is shown in the upper-right corner. When the reaction temperature (*T*) decreased, in the middle image, a broad molecular beam component was observed. Due to the low reaction temperature, most of the ketoketene, 6-carbonyl-2,4-cyclohexadien-1-one (*m*/*z* 120) **2**, was preserved and dissociatively photoionizes showing a fragmentation (CO loss) with kinetic energy release (a broad molecular beam component) to yield fragment ions at *m*/*z* 92. By increasing the sample concentration (right VMI), this feature becomes more apparent. Looking at the time-on-flight mass spectra, an asymmetric peak at *m*/*z* 92 was also observed, which is characteristic of fragmentation in the mass spectrometer, i.e., after ionization. To sum up, 6-carbonyl-2,4-cyclohexadien-1-one **2** (*m*/*z* 120) was fully decomposed to fulvenone **3** (*m*/*z* 92) at high temperatures while the former was preserved at lower ones, but still dissociatively photoionizes to fulvenone cations at $h\nu$ = 10.5 eV. To minimize the effect of dissociative ionization of 6-carbonyl-2,4-cyclohexadien-1-one (*m*/*z* 120) **2**, the temperature was optimized to fully decompose the precursor.

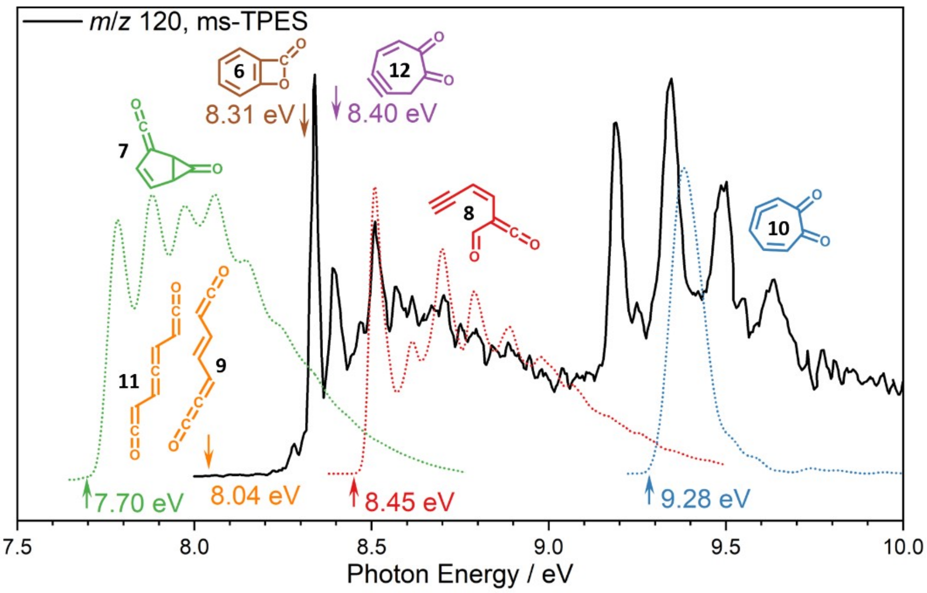

Figure S5. ms-TPES of *m*/*z* 120 with FC simulation of different isomers. The IE are calculated at G4 level of theory. The calculated FC envelopes and/or ionization energies of isomers **6–12** do not match to the features observed in the experimental ms-TPES, or could be ruled out due to thermodynamic considerations (see Figure 3).

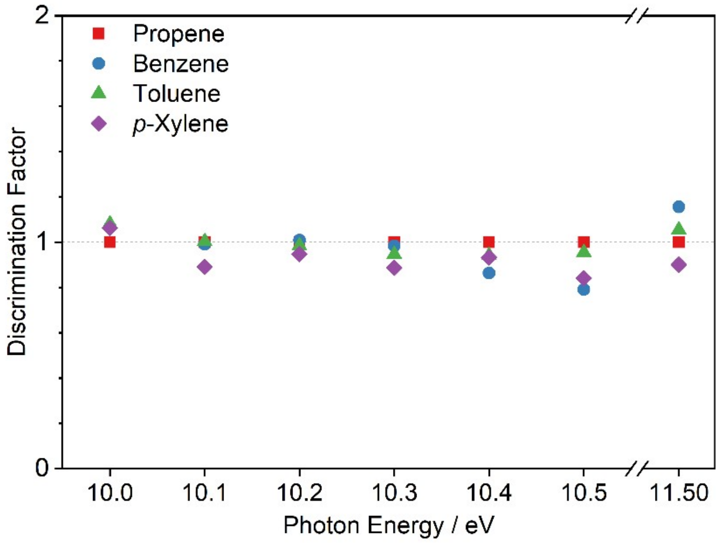

Figure S6. The mass discrimination factor of propene (*m*/*z* 42), benzene (*m*/*z* 78), toluene (*m*/*z* 92) and *p*-xylene (*m*/*z* 106) relative to propene.

In eq. 1 of the main text, the apparatus function comprises a mass-dependent element called mass discrimination factor. It is attributed to the flow distribution of molecules with different mass in the molecular beam and potentially different detection efficiencies as a function of mass.[9, 10] The velocity of all molecules along the beam axis is similar, while the velocity perpendicular to the beam axis scales with $1/\sqrt{m}$. Thereby, lighter have a broader distribution away from the molecular beam centerline, while oppositely the heavier species will be enriched in the centerline of the molecular beam expansion. When skimming the beam, the sampling probability is therefore higher for heavier molecules, which must be considered in the determination of the absolute photoionization cross section by introducing the mass discrimination factor. Mixtures of multiple components with exactly defined concentrations and known absolute photoionization cross section are used to determine the mass discrimination factor. Propene often serves as a reference due to its intermediate mass and well-known cross section. The mass discrimination factor then describes the ratio of the analytes' spectrometer response F_A to the one of propene F_P.

$$\frac{F_A}{F_P} = \frac{[P]}{[A]} \times \frac{\sigma_i^P}{\sigma_i^A} \times \frac{S_A}{S_P}$$

Here, S_A and S_P are the detected ion signals, $[A]$ and $[P]$ are the concentration of the two components in the gas mixture, and σ_i^A and σ_i^P are the photoionization cross sections of the analyte and propene, respectively. In our work, the mass discrimination factors of benzene, toluene and *p*-xylene were measured using propene as a reference. The results are depicted in Figure S4 and show that the mass discrimination factors scatter around unity, which agrees well with the results of Holzmeier *et al.*[11]

Table S3. PICS of fulvenone

Photon energy / eV	Fulvenone / Mb	Photon energy / eV	Fulvenone / Mb	Photon energy / eV	Fulvenone / Mb
8.00	0.1	8.86	18.5	9.72	29.0
8.02	0.1	8.88	19.3	9.74	28.8
8.04	0.1	8.90	19.3	9.76	27.9
8.06	0.2	8.92	20.0	9.78	28.2
8.08	0.2	8.94	20.4	9.80	28.1
8.10	0.2	8.96	21.0	9.82	27.5
8.12	0.3	8.98	21.9	9.84	27.8
8.14	0.3	9.00	22.4	9.86	27.1
8.16	0.4	9.02	22.7	9.88	26.7
8.18	0.5	9.04	23.4	9.90	26.5
8.20	0.5	9.06	23.7	9.92	25.9
8.22	0.7	9.08	24.5	9.94	26.0
8.24	1.7	9.10	24.5	9.96	25.4
8.26	2.1	9.12	25.2	9.98	25.1
8.28	2.4	9.14	25.5	10.00	24.8
8.30	2.8	9.16	25.9	10.04	24.4
8.32	3.2	9.18	26.1	10.06	25.0
8.34	3.5	9.20	26.2	10.08	24.4
8.36	4.0	9.22	26.6	10.10	24.6
8.38	4.7	9.24	26.8	10.12	24.1
8.40	5.3	9.26	27.4	10.14	23.9
8.42	6.0	9.28	28.3	10.16	23.6
8.44	6.7	9.30	27.8	10.18	23.9
8.46	6.8	9.32	27.6	10.20	23.3
8.48	7.6	9.34	28.5	10.22	23.1
8.50	8.2	9.36	28.2	10.24	22.7
8.52	9.3	9.38	28.5	10.26	22.7
8.54	10.1	9.40	28.6	10.28	21.9
8.56	10.3	9.42	28.4	10.30	22.0
8.58	10.7	9.44	28.9	10.32	21.2
8.60	11.4	9.46	29.7	10.34	21.1
8.62	11.8	9.48	29.0	10.36	21.0
8.64	12.2	9.50	28.9	10.38	20.6
8.66	13.1	9.52	29.1	10.41	20.4
8.68	13.8	9.54	29.0	10.43	20.2
8.70	14.2	9.56	29.0	10.44	19.5
8.72	14.7	9.58	28.8	10.46	18.7
8.74	15.2	9.60	29.1	10.48	18.8
8.76	16.1	9.62	29.7	10.50	18.8
8.78	16.4	9.64	29.8	10.52	18.6
8.80	17.0	9.66	29.4	10.54	17.8
8.82	17.4	9.68	29.3	10.56	17.9
8.84	18.0	9.70	29.1	10.58	17.9

Table S4. PICS of *m/z* 120 and 2-Carbonyl Cyclohexadienone

Photon energy / eV	*m/z* 120 / Mb	2-Carbonyl Cyclohexadie none / Mb	Photon energy / eV	*m/z* 120 / Mb	2-Carbonyl Cyclohexadie none / Mb
8.000	0.1	0.1	8.236	0.6	0.8
8.005	0.1	0.1	8.238	0.6	0.8
8.012	0.1	0.1	8.247	0.7	0.9
8.016	0.1	0.1	8.249	0.7	1.0
8.020	0.1	0.1	8.257	0.8	1.0
8.026	0.1	0.2	8.260	0.8	1.1
8.032	0.1	0.1	8.265	0.8	1.1
8.034	0.1	0.1	8.273	0.9	1.2
8.043	0.1	0.2	8.275	0.9	1.3
8.046	0.1	0.2	8.282	1.0	1.3
8.049	0.1	0.2	8.286	1.1	1.4
8.055	0.1	0.2	8.291	1.1	1.5
8.059	0.1	0.2	8.295	1.1	1.5
8.065	0.2	0.2	8.299	1.2	1.5
8.071	0.2	0.2	8.303	1.3	1.7
8.076	0.2	0.2	8.310	1.4	1.8
8.081	0.2	0.2	8.316	1.4	1.9
8.086	0.2	0.2	8.320	1.5	2.0
8.091	0.2	0.3	8.325	1.6	2.1
8.097	0.2	0.3	8.335	1.8	2.4
8.101	0.2	0.2	8.337	1.8	2.4
8.106	0.2	0.3	8.340	1.9	2.5
8.109	0.2	0.3	8.345	2.0	2.7
8.115	0.2	0.3	8.349	2.1	2.8
8.119	0.2	0.3	8.356	2.2	2.9
8.124	0.2	0.3	8.360	2.3	3.1
8.128	0.2	0.3	8.365	2.3	3.1
8.136	0.3	0.4	8.370	2.4	3.2
8.141	0.3	0.4	8.375	2.6	3.4
8.144	0.3	0.4	8.382	2.8	3.7
8.151	0.3	0.4	8.387	2.8	3.7
8.156	0.3	0.4	8.390	2.8	3.8
8.162	0.3	0.4	8.395	3.0	4.0
8.164	0.3	0.5	8.400	3.0	4.0
8.170	0.4	0.5	8.405	3.3	4.4
8.176	0.4	0.5	8.412	3.3	4.4
8.180	0.4	0.6	8.415	3.5	4.6
8.189	0.4	0.6	8.422	3.4	4.6
8.191	0.5	0.6	8.427	3.5	4.7
8.197	0.5	0.7	8.431	3.6	4.8
8.201	0.5	0.7	8.438	3.8	5.1
8.206	0.5	0.7	8.441	3.8	5.1
8.211	0.5	0.7	8.446	4.0	5.3
8.213	0.6	0.8	8.450	4.1	5.4
8.220	0.6	0.8	8.457	4.2	5.5
8.226	0.6	0.8	8.461	4.3	5.7
8.230	0.6	0.8	8.465	4.4	5.9

Continuing

Photon energy / eV	*m*/*z* 120 / Mb	2-Carbonyl Cyclohexadienone / Mb	Photon energy / eV	*m*/*z* 120 / Mb	2-Carbonyl Cyclohexadienone / Mb
8.469	4.5	5.9	8.704	9.6	12.8
8.475	4.6	6.2	8.709	9.6	12.8
8.479	4.7	6.3	8.716	9.7	12.9
8.485	4.7	6.3	8.719	9.8	13.1
8.490	5.0	6.6	8.723	9.8	13.0
8.496	5.1	6.8	8.732	9.9	13.2
8.501	5.1	6.8	8.736	9.9	13.3
8.505	5.2	7.0	8.741	9.9	13.4
8.511	5.3	7.2	8.746	10.0	13.5
8.512	5.5	7.4	8.751	10.1	13.5
8.521	5.5	7.5	8.757	10.2	13.7
8.525	5.7	7.7	8.761	10.2	13.7
8.530	5.7	7.7	8.765	10.4	13.8
8.533	5.8	7.8	8.771	10.5	13.9
8.541	6.1	8.2	8.775	10.5	14.0
8.546	6.2	8.2	8.780	10.4	14.1
8.551	6.4	8.5	8.785	10.6	14.2
8.555	6.5	8.6	8.792	10.7	14.4
8.559	6.5	8.6	8.795	10.8	14.4
8.566	6.7	8.9	8.801	10.8	14.5
8.570	6.7	9.1	8.806	10.9	14.6
8.573	6.9	9.2	8.812	10.9	14.6
8.579	7.0	9.4	8.816	11.0	14.7
8.585	7.2	9.5	8.820	11.0	14.8
8.591	7.4	9.8	8.824	11.1	14.8
8.596	7.5	10.0	8.830	11.2	15.0
8.600	7.6	10.1	8.835	11.3	15.1
8.605	7.8	10.4	8.839	11.4	15.2
8.611	7.9	10.5	8.846	11.5	15.3
8.615	7.9	10.6	8.851	11.6	15.5
8.620	7.9	10.6	8.856	11.5	15.3
8.626	8.0	10.7	8.858	11.4	15.2
8.631	8.0	10.8	8.863	11.5	15.4
8.634	8.1	11.0	8.870	11.6	15.6
8.642	8.2	11.1	8.876	11.7	15.6
8.649	8.4	11.3	8.880	11.5	15.4
8.645	8.5	11.4	8.885	11.8	15.7
8.654	8.6	11.6	8.890	11.7	15.9
8.659	8.6	11.6	8.895	11.8	15.9
8.664	8.6	11.6	8.900	11.9	16.0
8.669	8.8	11.8	8.905	12.0	16.2
8.674	8.9	11.9	8.910	11.9	16.1
8.680	9.0	12.1	8.915	12.0	16.2
8.687	9.2	12.2	8.918	12.0	16.2
8.690	9.3	12.4	8.928	12.1	16.3
8.695	9.3	12.5	8.930	12.1	16.4
8.701	9.5	12.7	8.936	12.1	16.4

Continuing

Photon energy / eV	*m*/*z* 120 / Mb	2-Carbonyl Cyclohexadienone / Mb	Photon energy / eV	*m*/*z* 120 / Mb	2-Carbonyl Cyclohexadienone / Mb
8.939	12.2	16.6	9.176	15.1	
8.946	12.3	16.7	9.181	15.2	
8.950	12.2	16.6	9.184	15.4	
8.957	12.2	16.6	9.190	15.5	
8.961	12.3	16.8	9.196	15.6	
8.965	12.4	16.8	9.203	15.7	
8.969	12.3	16.7	9.207	16.0	
8.974	12.4	16.8	9.209	15.9	
8.981	12.5	16.9	9.217	16.1	
8.985	12.5	16.9	9.220	16.2	
8.991	12.4	16.9	9.226	16.3	
8.995	12.5	17.0	9.232	16.4	
9.000	12.7	17.2	9.235	16.7	
9.004	12.7		9.240	16.6	
9.009	12.8		9.244	17.0	
9.016	12.9		9.249	17.1	
9.020	13.0		9.255	17.2	
9.024	13.1		9.260	17.2	
9.031	13.1		9.268	17.5	
9.037	13.1		9.269	17.4	
9.041	13.1		9.275	17.6	
9.046	13.3		9.278	17.8	
9.051	13.3		9.286	17.8	
9.055	13.3		9.290	17.8	
9.060	13.5		9.296	18.2	
9.065	13.4		9.301	18.2	
9.068	13.6		9.304	18.3	
9.077	13.6		9.312	18.6	
9.079	13.8		9.317	18.6	
9.085	13.9		9.322	18.8	
9.090	14.0		9.328	19.0	
9.095	14.0		9.330	19.0	
9.100	14.1		9.335	19.2	
9.106	14.2		9.342	19.3	
9.111	14.3		9.346	19.6	
9.115	14.1		9.350	19.6	
9.119	14.3		9.356	19.8	
9.125	14.3		9.361	19.8	
9.129	14.3		9.365	19.9	
9.134	14.5		9.367	20.2	
9.142	14.6		9.377	20.3	
9.144	14.7		9.380	20.5	
9.150	14.8		9.386	20.9	
9.154	14.9		9.390	20.8	
9.160	15.1		9.395	20.9	
9.165	15.1		9.398	20.9	
9.173	15.0		9.406	21.0	

Continuing

Photon energy / eV	*m*/*z* 120 / Mb	2-Carbonyl Cyclohexadienone / Mb	Photon energy / eV	*m*/*z* 120 / Mb	2-Carbonyl Cyclohexadienone / Mb
9.409	20.9		9.644	26.5	
9.414	21.2		9.650	26.8	
9.422	21.3		9.657	26.9	
9.431	21.4		9.661	26.8	
9.432	21.6		9.664	26.9	
9.436	21.6		9.672	27.1	
9.441	21.7		9.675	26.9	
9.445	21.8		9.680	27.1	
9.449	21.9		9.687	27.2	
9.456	21.9		9.692	27.1	
9.460	22.1		9.697	27.1	
9.467	22.4		9.699	27.1	
9.469	22.4		9.706	27.3	
9.475	22.6		9.713	27.4	
9.479	22.7		9.715	27.5	
9.486	22.9		9.721	27.5	
9.491	23.1		9.728	27.5	
9.494	23.2		9.730	27.6	
9.502	23.3		9.735	27.5	
9.504	23.5		9.740	27.6	
9.508	23.3		9.749	27.2	
9.516	23.7		9.757	27.7	
9.521	24.1		9.756	27.6	
9.521	24.1		9.766	27.7	
9.530	24.2		9.769	27.6	
9.535	24.4		9.776	27.6	
9.540	24.4		9.780	27.6	
9.545	24.4		9.784	27.8	
9.554	24.8		9.791	27.9	
9.558	24.7		9.797	27.8	
9.561	24.8		9.799	28.0	
9.565	24.9		9.804	28.1	
9.570	25.1		9.811	28.0	
9.576	25.3		9.817	28.2	
9.579	25.1		9.819	28.2	
9.587	25.5		9.826	28.2	
9.591	25.5		9.831	28.2	
9.593	25.5		9.835	28.2	
9.601	25.6		9.842	28.1	
9.607	25.9		9.845	28.2	
9.611	25.9		9.850	28.3	
9.615	25.9		9.853	28.3	
9.622	26.0		9.859	28.5	
9.627	26.2		9.866	28.5	
9.631	26.2		9.871	28.4	
9.636	26.4		9.874	28.3	
9.641	26.5		9.882	28.5	

Continuing

Photon energy / eV	*m*/*z* 120 / Mb	2-Carbonyl Cyclohexadienone / Mb
9.886	28.5	
9.889	28.5	
9.898	28.5	
9.900	28.5	
9.907	28.4	
9.908	28.5	
9.914	28.6	
9.919	28.6	
9.924	28.7	
9.930	28.7	
9.937	28.6	
9.941	28.6	
9.945	28.5	
9.949	28.5	
9.956	28.6	
9.960	28.4	
9.964	28.4	
9.974	28.4	
9.971	28.4	
9.979	28.3	
9.987	28.3	
9.989	28.2	
9.995	28.3	
9.999	28.3	
10.052	28.1	
10.098	28.5	
10.152	29.0	
10.191	29.2	
10.248	29.9	
10.301	30.4	
10.351	30.7	
10.398	30.9	
10.453	31.2	
10.503	31.2	

Reference

1. T. P. Debies and J. W. Rabalais, *J. Am. Chem. Soc.*, 1975, **97**, 487-492.
2. J. P. Maier, *Angew. Chem. Int. Ed.*, 1981, **20**, 638-646.
3. G. Bieri, F. Burger, E. Heilbronner and J. P. Maier, *Helv. Chim. Acta*, 1977, **60**, 2213-2233.
4. G. Bieri, J. D. Dill, E. Heilbronner, J. P. Maier and J. L. Ripoll, *Helv. Chim. Acta*, 1977, **60**, 629-637.
5. D. Kaiser, E. Reusch, P. Hemberger, A. Bodi, E. Welz, B. Engels and I. Fischer, *Phys. Chem. Chem. Phys.*, 2018, **20**, 3988-3996.
6. H. Bock, T. Hirabayashi and S. Mohmand, *Chem. Ber.*, 1981, **114**, 2595-2608.
7. R. Schulz and A. Schweig, *Tetrahedron Lett.*, 1979, **20**, 59-62.
8. P. Hemberger, Z. Pan, A. Bodi, J. A. van Bokhoven, T. K. Ormond, G. B. Ellison, N. Genossar and J. H. Baraban, *ChemPhysChem*, 2020, **21**, 2217-2222.
9. J. B. Howard, *Massachusetts Institute of Technology,* , 1981, **1**.
10. D. Rösch, R. L. Caravan, C. A. Taatjes, K. Au, R. Almeida and D. L. Osborn, *J. Chem. Phys. A*, 2021, **125**, 7920-7928.
11. F. Holzmeier, I. Fischer, B. Kiendl, A. Krueger, A. Bodi and P. Hemberger, *Phys. Chem. Chem. Phys.*, 2016, **18**, 9240-9247.
